# Regulating the size of antimony nanoparticles to enhance the photo-response in the near-infrared region and anti-hepatoma cell activity

**DOI:** 10.3389/fbioe.2025.1656253

**Published:** 2025-09-05

**Authors:** Lingling Huang, Yimin Gong, Zhijian Chen, Yanjun Tan, Qian Gao, Yilei Wang, Yuyu Gao, Wanting Cheng, Weiyuan Liang, Xiaoli Yang

**Affiliations:** ^1^ School of Pharmacy, Guangxi Medical University, Nanning, Guangxi, China; ^2^ Scientific Research Center, Guilin Medical University, Guilin, Guangxi, China; ^3^ Guangxi Health Commission Key Laboratory of Disease Proteomics Research, Guilin, Guangxi, China; ^4^ Department of Chemistry, Fudan University, Shanghai, China; ^5^ Key Laboratory of Clinical Laboratory Medicine of Guangxi Department of Education, Nanning, China; ^6^ Department of Clinical Laboratory, The First Affiliated Hospital of Guangxi Medical University, Nanning, China

**Keywords:** antimony nanoparticles, SPR, photothermal therapy, photodynamic therapy, hepatoma cell

## Abstract

**Introduction:**

Antimony (Sb) has been used as a medication for centuries, while it has rarely been investigated in plasmonic phototherapy, partly due to the lack of effective liquid-phase controllable synthesis methods to construct Sb nanocrystals with an optimized absorption curve within the biological transparent window (near-infrared region), achieving more effective and less side-effect phototherapy.

**Methods:**

Herein, an effective ligand-guided growth strategy was employed to synthesize Sb nanoparticles (Sb NPs) with high photothermal conversion efficiency (PTCE). The spatial electric field distribution of Sb NPs was simulated by the finite-difference time-domain (FDTD) method to validate their localized surface plasmon resonance (LSPR) effect. Sb NPs were coated with polydopamine (PDA) and polyethylene glycol (PEG) to enhance their biocompatibility. The synergistic anti-hepatoma activities of Sb NPs were evaluated via *in vitro* experiments.

**Results:**

Sb NPs were successfully obtained via a ligand-guided growth strategy. Uv-vis absorption peak was observed to red-shift from 520 nm to 810 nm as the size of Sb NPs increased from 40 nm to 70 nm. Sb NPs achieve a PTCE of 59.3% under 808 nm resonant excitation and was favorable to photothermal therapy (PTT). Sb NPs also exhibit 660 nm laser responsiveness, producing reactive oxygen species (ROS) that enable photodynamic therapy (PDT). *In vitro* anti-BEL-7404 hepatoma cells experiments revealed that 660 nm/808 nm laser irradiation could inhibit proliferation, promote apoptosis, and induce G2/M phase blockage tendency, with combined irradiation exhibiting more significant effects.

**Conclusion:**

The fabricated Sb-PDA exhibits synergistic PTT/PDT potential, though its *in vivo* efficacy and mechanisms warrant deeper investigation. LSPR-induced Sb-based nanomedicine may unlock diverse biomedical applications of semimetals.

## 1 Introduction

Hepatoma ranks as the sixth most prevalent malignancy worldwide and represents the third leading cause of cancer mortality (Bray et al., 2024). This disease typically presents with nonspecific early symptoms, demonstrates unfavorable clinical outcomes, and exhibits elevated fatality rates. Current therapeutic approaches primarily include surgical intervention, radiation therapy, systemic chemotherapy, and organ transplantation. Transplantation yields a 5-year survival rate approaching 80%, whereas resection achieves only approximately 50% survival (Gentile et al., 2019). These outcomes underscore the urgent need for novel treatment modalities against this life-threatening condition (Lu et al., 2022).

In recent years, optical therapies combined with novel photoactive materials have attracted attention in innovative cancer therapies. PTT emerged as a new tumor treatment strategy that has been widely studied due to its minimally invasive nature and specific targeting capability for tumor cells (Kumar et al., 2021). The PTT process uses photothermal therapeutic agents (PTAs) to generate localized heating effects to kill poorly vascularized cancer cells (Zhou et al., 2019), while using near infrared (NIR) light as a remote stimulation method to achieve highly spatial and temporal control during localized heating, minimizing side effects (Hussein et al., 2018). To enhance the effectiveness of tissue heating, it is necessary to minimize tissue absorption and scattering, thereby increasing light penetration depth (Ding et al., 2014). Strong absorption is the primary parameter to ensure high photothermal performance. On the other hand, NIR operates stably, can be locally focused on specific areas, and can more effectively penetrate biological tissues such as skin and blood, belonging to the optical transparent window of biological tissues (Smith et al., 2009). A large number of studies have been dedicated to developing new agents with high PTCE (Cao et al., 2020). However, optimizing the absorption curve of materials within the biological transparent window through appropriate regulation methods, constructing materials with enhanced photothermal effects in the NIR region, and achieving more effective and less side-effect photothermal therapy remain challenging, representing a key scientific issue in the application of photothermal therapy.

Although extensive experimental and theoretical research has been conducted on noble metal and semiconductor nanocrystals in the field of PTT driven by LSPR, and it has been confirmed that adjusting the doping ratio, photochemical modulation, or electrochemical modulation can extend the LSPR absorption range from the visible light region to the NIR region, they still face issues such as a narrow natural resonance frequency range, insufficient morphological thermal stability, NIR absorption loss, and the need to improve PTCE (Chen et al., 2019; Zheng et al., 2024; Lei et al., 2023). In contrast, research on semi-metallic nanomaterials in the field of PTT has not received sufficient attention, and their potential applications in this field still require in-depth exploration.

Arsenic, antimony, bismuth and other metalloid/semiconductor materials, owing to their unique structures and extraordinary electronic properties, have exhibited extensive application potential in fields such as optoelectronics and energy field (He et al., 2014; Guo et al., 2021). Antimony has been used as a medication for centuries, long employed in the treatment of leishmaniasis and schistosomiasis, with its pentavalent compounds remaining first-line therapeutic agents for leishmaniasis to date (Frézard et al., 2009). In recent years, 2D antimony (antimonene) have emerged as a new type of PTAs and attracted widespread attention (Tao et al., 2017; Peng et al., 2019; Zhang et al., 2020). These materials exhibit excellent PTCE attributed to their strong NIR absorption capacity. However, further enhancing PTCE remains challenging, despite Sb NPs theoretically possessing plasmonic properties and exhibiting stronger NIR absorption than gold (Au) nanomaterials (Torres et al., 2021), the regulatory effects of different geometric configurations on the LSPR of Sb NPs still lack systematic experimental investigation (Torres et al., 2021; Chen et al., 2021; Chen et al., 2022). Therefore, the development of morphology-controllable preparation methods to fabricate high-quality Sb NPs with tunable LSPR properties holds significant importance for the development of novel PTA with high PTCE.

**SCHEME 1:**
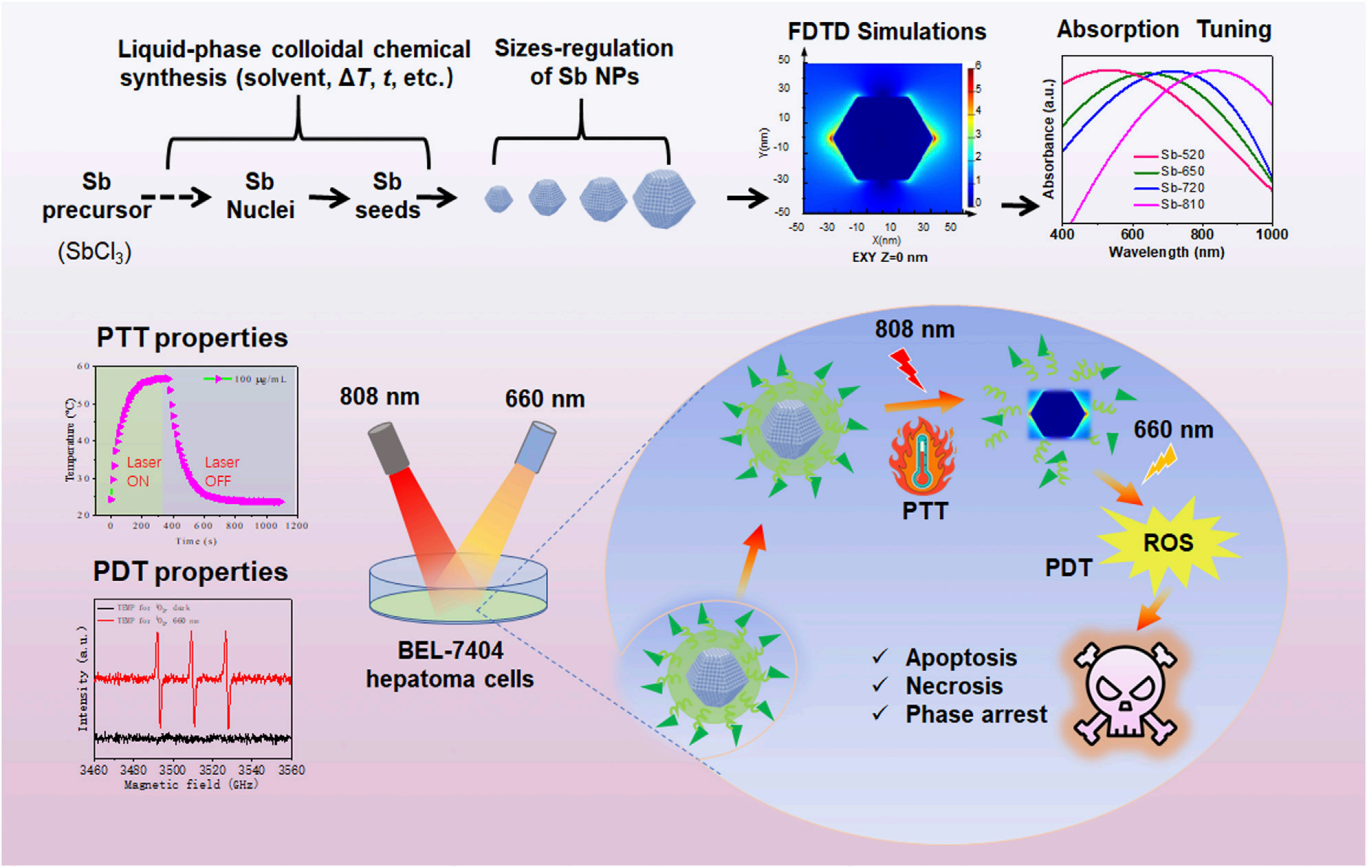
Schematic diagram of regulating the SPR-related absorption curve of Sb NPs to enhance photothermal therapy (PTT) and photodynamic (PDT) therapies in BEL-7404 hepatoma cells.

Herein, we employed a modified ligand-guided growth strategy proposed by Chen et al. (2021) to controllably synthesize Sb NPs. The size of Sb NPs could be adjusted by regulating the reaction temperature, the amount of SbCl_3_, and the molar ratio of dodecanethiol (DDT) to oleylamine (OLA). To enhance biocompatibility, Sb NPs were modified with PDA and folic acid-modified PEG. Experimentally, the modulation effect of different sizes on the LSPR of Sb NPs was successfully observed. As the size of Sb NPs increased from 40 nm to 70 nm, the UV-vis absorption peak was observed to red-shift from 520 nm to 810 nm. When the SPR resonance frequency matched the external excitation light (808 nm), the PTCE of Sb NPs reached up to 59.3%. Sb NPs also exhibited sensitivity to 660 nm laser and generated that enable photodynamic therapy (PDT). *In vitro* anti-BEL-7404 hepatoma cells experiments revealed that 660 nm/808 nm laser irradiation could inhibit proliferation, promote apoptosis, and induce G2/M phase blockage tendency, with combined irradiation exhibiting more significant effects. This LSPR-induced antimony-based nanodrug may stimulate the various potential applications of semimetals in biomedicine (Scheme 1).

## 2 Experimental section

### 2.1 Materials

Antimony trichloride (SbCl_3_, 99.999%) was purchased from Macklin. Dodecylthiol (DDT, 99.9%), Oleylamine (OLA, 70%), Oleic acid (OA, 90%), technical grade octadecene (ODE, 90%), tert-Butylamine borane (99%), Dopamine hydrochloride, 1,3-Diphenylisobenzofuran and Propidium Iodide were purchased from Sigma Aldrich. Toluene, absolute ethanol, and dichloromethane (DCM) were obtained from Xilong Scientific Co., Ltd., China. Folic acid PEG was purchased from Xi’an ruixi Biological Technology co., Ltd., China. Glutaric dialdehyde, Uranium acetate and Lead citrate were provided by TED PELLA, Inc., United States of America. The high-sugar medium, phosphate buffer solution and penicillin/streptomycin mixed solution were purchased from Gibco Company of the United States. Fetal bovine serum is from Lonsera. Unless otherwise mentioned, all chemicals were used as received without further purification.

### 2.2 Cells and culture

All the cell lines were purchased from the Cell Bank of the Chinese Academy of Sciences. The L-O2 (normal liver cells), HepG2 (human hepatoma cells) and BEL-7404 (human hepatoma cells) cell lines were cultured with DMEM (Sangon Biotech, E600003-0500). The cells were incubated with 5% CO_2_ at 37 °C for cultivation.

### 2.3 Synthesis of Sb NPs

Sb NPs was prepared by a modified ligand-guided growth method proposed by Chen (Refer to: Adv. Mater., 2021, 33 (18): 2100039). Specifically, under nitrogen protection, SbCl_3_, DDT, ODE and OLA were added successively to the three-necked flask, among which DDT and OLA were used as capping agents. Then tert-butylamine methylborane was added as a reducing agent to trigger the growth process of Sb NPs. Adjusting the reaction temperature, the molar number of SbCl_3_ and the molar ratio of DDT/OLA can regulate the morphology of Sb NPs.

To study the detailed reaction kinetic in the synthesis protocol for Sb NPs, we conduced TEM morphological analysis at different reduction duration time (0 s, 10 s, 30 s, 180 s, 10 min, 20 min, 30 min).

### 2.4 Modification of Sb NPs

Dopamine self-assembly was used to form PDA nanospheres encapsulating Sb NPs. Subsequently, amine (-NH_2_)-terminated PEG (i.e., FA-PEG-NH_2_) was employed to modify Sb-PDA nano-compounds via Michael addition reaction or Schiff base reaction, aiming to enhance their mono-dispersity and cellular uptake (RSC Advances, 2017, 7 (63): 39641–39650.). After modification, the samples were label as Sb-PDA.

### 2.5 Physicochemical properties characterization of Sb NPs

Morphologies of Sb NPs were examined by using a TEM (HT7700, Hitichi, Japan). Software of Nano Measurer (version 1.2.5) was employed to statistical analysis of the particle size distribution based on the TEM images. The ultraviolet absorption spectra of Sb NPs were obtained by using the UV-2401PC ultraviolet-visible spectrophotometer produced by Shimadzu Corporation of Japan. Zeta potential of NSs were performed by Zetasizer Nano ZS90 (Malvern Instruments Ltd., United Kingdom). The types of ROS in Sb NPs were determined and quantified by electron paramagnetic spin resonance (ESR). The distribution of the four elements C, N, O and Sb in the Sb-PDA composite material was determined by energy dispersive spectrometer (EDS).

### 2.6 FDTD simulation

The spatial electric field distribution of as-prepared Sb NPs under incident laser (808 nm or 660 nm) was simulated by a modified FDTD method (Refer to Adv. Mater., 2021, 33 (18): 2100039 and Nat. Commun., 2019, 10 (1): 28.). In the FDTD simulations, the Sb NPs with diameter 65 nm and thickness 65 nm which was abbreviated as Sb-810. The mesh grid near the structure was set as 1 nm.

### 2.7 Photothermal performance measurement of Sb NPs

Ultrapure water as used as the blank control group. Gradience concentrations (6.25, 12.5, 25, 50, and 100 μg/mL) of Sb NPs or Sb-PDA solutions were prepared. Take 1 mL of each solution into a quartz dish to detect their photothermal properties and calculate the PTCE, respectively. The parameters of the NIR laser were set as follows: wavelength of 808 nm and power density of 1.5 W/cm^2^. Data were recorded every 1 s, with a total irradiation time of 1100 s.

### 2.8 Cellular experiments

#### 2.8.1 Cell viability

Cell viability was determined by using a CCK-8 assay kit. HepG2, BEL-7404, and L-O2 cells were seeded in 96-well plates at a density of 1 × 10^4^ cells per well. After 24 h of incubation, cells were exposed to Sb-PDA composites at different dose levels (0, 6.25, 12.5, 25, and 50 μg/mL) and incubated for an additional 24 h. Each group included 5 replicate wells, and the experiment was repeated 3 times.

#### 2.8.2 Cell proliferation experiment

The experiment was divided into four groups: (1) Blank (without material), (2) 808 nm laser treatment (808 nm near-infrared irradiation at 1.5 W/cm^2^ for 10 min), (3) 660 nm laser treatment (660 nm laser irradiation at 0.5 W/cm^2^ for 10 min), and (4) 660 nm + 808 nm laser treatment group (808 nm laser at 1.5 W/cm^2^ and 660 nm laser at 0.5 W/cm^2^ separately irradiated for 10 min each). BEL-7404 cells were first co-incubated with 50 μg/mL Sb-PDA nanocomposites for 4 h, followed by 10 min of different laser irradiation treatments. After subsequent 12-h incubation, cell viability was detected using the CCK-8 assay kit.

#### 2.8.3 Pretreatment of cells for TEM

BEL-7404 cells were treated and collected with 2.5% glutaraldehyde by semi-in situ immobilization. After stored at 4 °C overnight, the samples were sequentially washing, post-fixing, block staining, gradient ethanol and acetone dehydration, infiltrated and polymerization with epoxy resin Epon 812. Ultrathin sections were double-stained with uranyl acetate and lead citrate, and observed under a Hitachi HT7700 (Hitachi, Japan) TEM at an accelerating voltage of 80 kV.

#### 2.8.4 Staining of dead/living cells

The groups were set as follows: blank group (with material), 808 nm laser treatment group (808 nm near-infrared irradiation at 1.5 W/cm^2^ for 10 min), 660 nm laser treatment group (660 nm laser irradiation at 0.5 W/cm^2^ for 10 min), and 660 nm + 808 nm laser treatment group (808 nm laser at 1.5 W/cm^2^ and 660 nm laser at 0.5 W/cm^2^ separately irradiated for 10 min each). BEL-7404 cells in the logarithmic growth phase were seeded in 96-well plates at a density of 1 × 10^4^ cells/well, with three replicate wells per group. After adding 50 μg/mL Sb-PDA composites and co-incubating with cells for 4 h, cells underwent different laser treatments followed by an additional 12-hour co-incubation. The medium was gently aspirated, and 100 μL PBS, 1.2 μL Calcein-AM, and 6 μL PI were added to each well. After 15 min incubation, cells were observed under an inverted fluorescence microscope.

#### 2.8.5 Cell apoptosis experiment

According to the kit instructions, 100 μL of 1×Binding buffer was added to each tube to gently resuspend and mix the cells. Subsequently, 5 μL of Annexin V-PE was added and mixed gently by pipetting. Next, 5 μL of 7-AAD staining solution was added and mixed again. The samples were incubated at room temperature protected from light for 15 min. After incubation, 400 μL of 1 × Binding buffer was supplemented to each tube and mixed gently. The cell suspension was filtered through a 400-mesh sieve into flow cytometry tubes, vortexed, and then analyzed using a flow cytometer to detect cell apoptosis. Prepared samples were subjected to detection within 1 h.

#### 2.8.6 Cell cycle experiment

Cells were digested with 0.25% EDTA-trypsin. After digestion, 2 mL of complete DMEM medium was added to terminate digestion. The cell suspension was pipetted to mix thoroughly and transferred to corresponding 15 mL centrifuge tubes for each group. Centrifugation was performed at 2000 rpm for 15 min. The supernatant was discarded, and 500 μL of 70% cold ethanol was added to fix the cell pellet, followed by storage at 4 °C overnight. The next day, pre-cooled PBS solution was added, and centrifugation at 2000 rpm was conducted for 5 min. After discarding the supernatant, pre-cooled PBS was added again to wash once, followed by centrifugation to remove the fixative. Then 500 μL of PI/RNase staining buffer was added, pipetted to mix thoroughly, and incubated at 37 °C protected from light for 15 min. Finally, the cell suspension was filtered through a 400-mesh sieve into flow cytometry tubes for detection.

### 2.9 Statistical analysis

Biological replicates were done in all experiments unless otherwise stated. Student’s t-test were used to analysis the significant different of the data. The SPSS software 19.0 were used to perform statistical analysis. And the p value < 0.05 was considered statistically significant.

## 3 Results and discussion

### 3.1 Size-controlled synthesis and characterization of Sb NPs for NIR photo-response tuning

**FIGURE 1:**
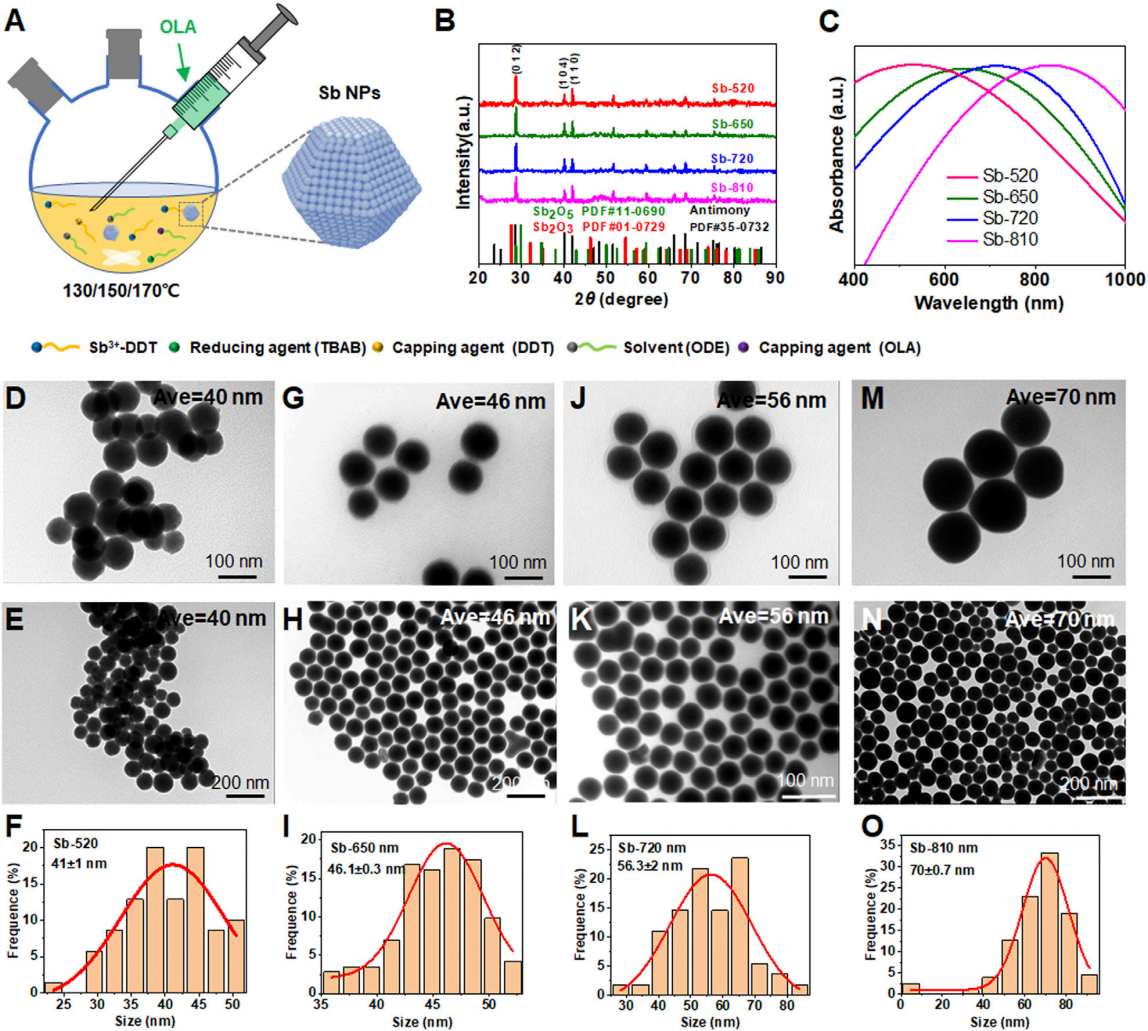
Size-controlled synthesis and characterization of Sb NPs for near-infrared photo-response tuning. **(A)** Fabrication schematic of Sb NPs. **(B)** XRD patterns and **(C)** Vis-NIR absorption spectra of four Sb NP samples (labeled Sb-520, Sb-650, Sb-720, Sb-810 according to UV-Vis absorption peaks at 520, 650, 720, and 810 nm, respectively). TEM analysis: Sb-520 **(D)** local view, **(E)** global view, **(F)** size distribution histogram from **(E)**; Sb-650 **(G)** local view, **(H)** global view, **(I)** size distribution from **(H)**; Sb-720 **(J)** local view, **(K)** global view, **(L)** size distribution from **(K)**; Sb-810 (**(M)** local view, **(N)** global view, **(O)** size distribution from **(N)**.

The nucleation and growth of crystals refer to the self-assembly of smaller structural units (such as metal atoms) through weak interactions to form relatively complex nanostructures, typically including epitaxial growth and liquid-phase synthesis methods (Xu et al., 2020). Among these, liquid-phase synthesis enables the preparation of highly uniform nanoparticles due to the homogeneous mixing of precursors at the molecular level, while also allowing precise control over structural parameters. Relevant theories for liquid-phase synthesis include classical crystal growth theory (LaMer and Theory, 1950), Wulff’s free energy theory (Wulff, 1901), and controllable synthesis of metal nanoparticles through thermodynamic control, kinetic control, and oxidative etching (Chen et al., 2022; Xia et al., 2009). Notably, the ligand-directed growth strategy has proven particularly effective for synthesizing Sb NPs (Chen et al., 2021). The schematic diagram of the experimental setup for the controllable size synthesis of Sb NPs is shown in Figure 1A. Time-resolved TEM morphological analysis revealed that Sb NPs with particle sizes exceeding 20 nm were formed within 10 s of the reduction reaction; by 3 min, the particle distribution became more uniform (average particle size ∼40 nm), and continued growing to 180 nm at 20 min. Interestingly, at 30 min of reaction, no further particle size increase was observed (showing size heterogeneity), accompanied by increasingly irregular morphology, suggesting possible degradation of the sample (Supplementary Figure S2) (Chen et al., 2021; Chen et al., 2022).

Then, Figures 1B–O and Supplementary Figure S1 systematically characterizes the morphology, crystal structure, and optical properties of Sb NPs synthesized under varied conditions. XRD patterns (Figure 1B) confirm all samples crystallize in the rhombohedral Sb phase (JCPDS 35–0732), with no detectable diffraction peaks for Sb_2_O_3_ or Sb_2_O_5_, indicating minimal oxidation. UV-Vis spectra (Figure 1C) demonstrate morphology-dependent localized surface plasmon resonance (LSPR) modulation: as particle size progressively increases from 40 nm to 70 nm (Figures 1D–O), the absorption peak red-shifts sequentially from 520 nm to 810 nm. This size evolution correlates directly with critical growth kinetics parameters including temperature, duration, SbCl_3_ concentration and dodecanethiol (DDT)/oleylamine (OLA) molar ratio (LaMer and Theory, 1950; Wulff, 1901; Xia et al., 2009). The phenomenon of the UV-vis absorption peak of Sb NPs red-shifting with the increase in size may be attributed to the combined action of size-dependent electron oscillation characteristics and dielectric environment regulation, whose theoretical basis can be attributed to Mie theory (classical electromagnetic theory) and the LSPR model (Dorodnyy et al., 2023; Yang et al., 2021).

### 3.2 FDTD simulation, PTT and PDT properties of Sb NPs

**FIGURE 2:**
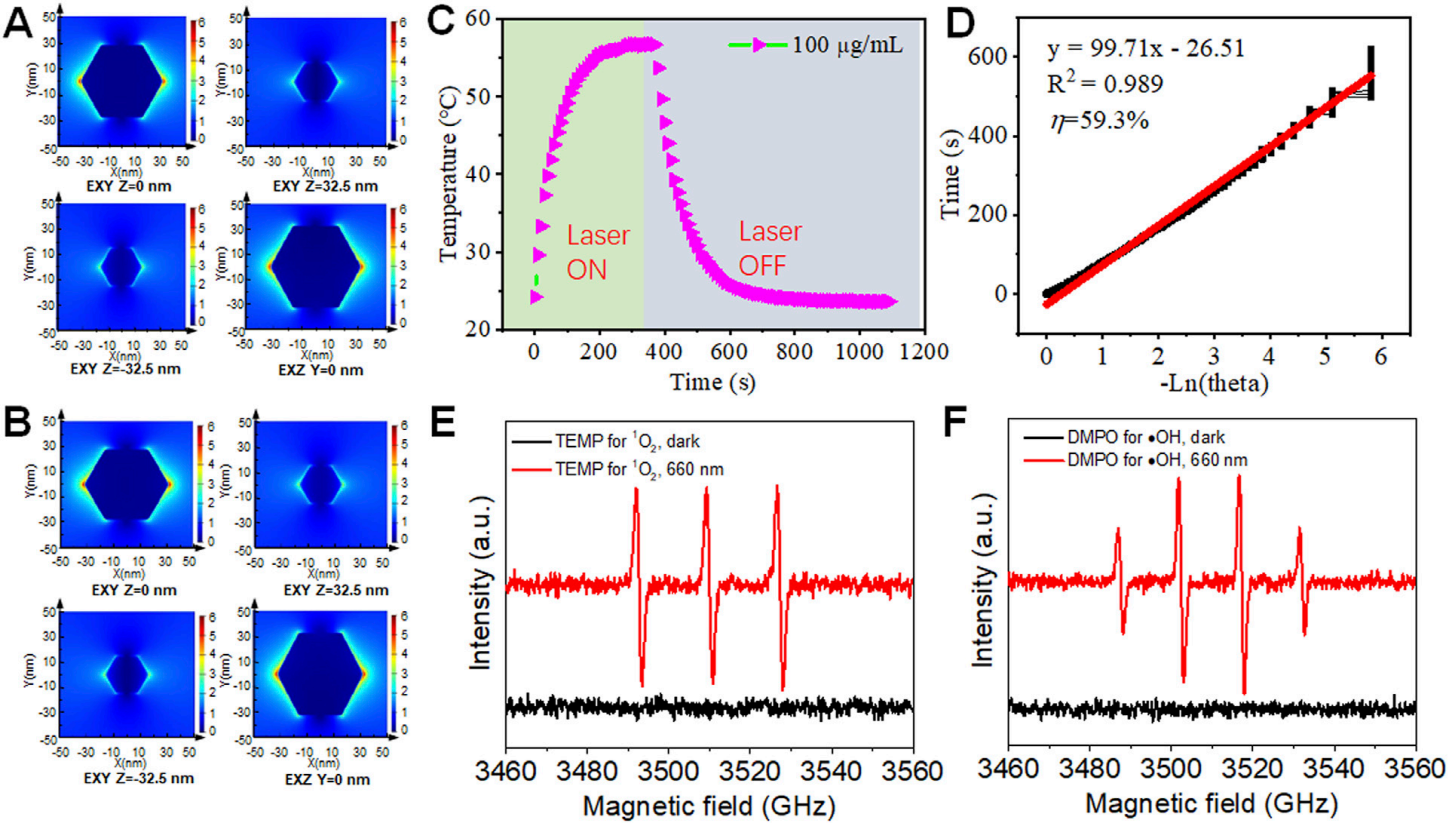
FDTD simulation, PTT and PDT properties of Sb NPs. Spatial electric field distribution of a single Sb NPs with the diameter and height (thickness) of 65 nm under 808 nm **(A)** and 660 nm **(B)** laser illumination calculated by FDTD. **(C)** Photothermal warming (laser on), cooling (laser off) curves of Sb NPs. **(D)** Calculation photothermal efficiency of Sb NPs. **(E)** Electron paramagnetic spin resonance (ESR) spectra of Sb NPs for detecting singlet oxygen **(E)** and hydroxyl radicals **(F)**.

The spatial electric field distribution of Sb NPs (Sb-810) with a maximum absorption peak at 810 nm under 808 nm or 660 nm excitation incidence light source was simulated using the FDTD method to validate their LSPR effect (Chen et al., 2021; Xue et al., 2019). As shown in Figure 2A, sub-diffraction-limit electromagnetic field localization was observed at the interface under 808 nm excitation, with high field intensity concentrated at geometric tips, indicating a curvature-dependent field enhancement property inherent to sharp structures. Since 660 nm lasers are commonly used in photodynamic therapy, the electric field distribution of Sb-810 was further simulated under 660 nm incidence light source (Figure 2B). The field distribution characteristics were similar to those under 808 nm excitation, confirming that 660 nm light can effectively activate the LSPR response of Sb-810. These results have laid the theoretical foundation for enhancing the photothermal performance of Sb NPs under 808 nm laser irradiation and advancing research on 660 nm laser-driven photodynamic therapy.

Based on the aforementioned research foundation, we systematically validated the photothermal and photodynamic performance of Sb NPs. Photothermal performance tests demonstrated that according to the heating-cooling curves under 808 nm laser irradiation (Figures 2C,D), the PTCE of Sb-PDA composites in aqueous solution reached 59.3%, which was significantly higher than most reported photothermal agents (Supplementary Table S1), confirming their efficient NIR photothermal efficacy. Photodynamic performance was characterized by electron spin resonance (ESR) spectroscopy and chemical probe. Under 660 nm laser irradiation, the TEMP trapping system exhibited a characteristic 1:1:1 triplet ESR signal (Figure 2E), confirming singlet oxygen (^1^O_2_) generation (Konaka et al., 1999). The DMPO trapping system displayed a 1:2:2:1 quartet signal (Figure 2F), verifying hydroxyl radical (·OH) production (Cao et al., 2018). DPBF probe experiments further revealed significant attenuation of the characteristic absorption peak at 410 nm after 660 nm laser irradiation (Supplementary Figure S3), indicating high singlet oxygen yield. These results collectively demonstrate that Sb NPs possess dual functionality for synergistic PTT and PDT (Ouyang et al., 2025).

### 3.3 Modification and stability of Sb NPs

**FIGURE 3:**
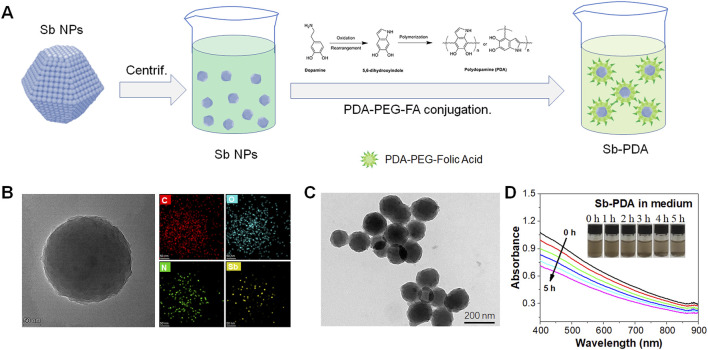
Modification and stability of Sb NPs. **(A)** Schematic illustration of Sb NPs sequentially coated with polydopamine (PDA) and folic acid-modified polyethylene glycol (FA-PEG). **(B)** TEM morphology and EDS mapping of Sb-PDA composites. **(C)** TEM morphology of Sb-PDA composites. **(D)** UV absorption spectroscopic curves of the stability of composites.

Biocompatibility is crucial for the application of nanoparticles in cell biology. To enhance the biocompatibility of Sb NPs, a PDA and FA-PEG dual-coating modification strategy was employed (Figure 3A). Results in Figures 3B,C demonstrate that modified Sb NPs form spherical Sb-PDA composite particles with uniform size of approximately 200 nm. EDS mapping confirms the homogeneous distribution of C, N, O, and Sb elements (Figure 3B), indicating successful encapsulation of Sb NPs by PDA layer.

In PDA coating systems, the size effect where the UV-vis absorption peak induced by LSPR redshifts with increasing Sb NP size remains applicable but is modulated by the dielectric environment and interfacial interactions introduced by the PDA coating. This can be interpreted as followings. 1) Physical basis of the size effect. The LSPR peak position of metallic Sb NPs is primarily determined by their size, shape, and dielectric environment. As size increases, the spatial expansion of electron oscillations leads to a reduction in resonance frequency (redshift), and this classical electromagnetic response mechanism persists in PDA-coated systems (Lermé et al., 2017). For example, the longitudinal LSPR peak of gold nanorods exhibits significant redshift with increasing aspect ratio, even in the presence of surface ligands or coatings (He et al., 2020). 2) Dielectric screening effect of the PDA coating. The high refractive index (∼1.7) of PDA alters the local dielectric constant around the nanoparticles, causing an overall redshift of the LSPR peak. Studies show that a 20 nm-thick PDA coating can redshift the LSPR peak of gold nanoparticles by approximately 18–73 nm (depending on the initial peak position), while the size-dependent peak shift trend is retained (Hou et al., 2020). The dielectric properties of PDA modify the resonance condition but do not completely suppress the size effect (Lermé et al., 2017). 3) Coating thickness and distance dependence. As a rigid spacer layer, PDA enables precise control of the fluorophore-metal surface distance (5–25 nm). When the coating thickness is fixed, increasing nanoparticle size still induces redshift; however, if the PDA thickness increases simultaneously, near-field coupling may weaken, partially offsetting the size effect (Hou et al., 2020). In summary, PDA-coated systems retain the fundamental principles of LSPR size effects but require consideration of dielectric environment adjustments and distance regulation. This characteristic is particularly critical for biosensing and photothermal therapy design.

To evaluate the stability of Sb NPs in solution before and after surface modification, zeta potential analysis were performed. It is generally considered that particles with zeta potentials greater than +30 mV (positive) or less than −30 mV (negative) exhibit good stability in solution (Hunter, 1981; Deng et al., 2023; Luo et al., 2020). As shown in Supplementary Figure S4, the zeta potential of unmodified Sb NPs was −43.1 mV, which further decreased to −49.5 mV after modification, indicating that Sb NPs maintained good stability both before and after modification, with surface modification further enhancing their stability. UV-vis absorption measurements of Sb-PDA in culture medium were conducted to assess long-term stability. As demonstrated in Figure 3D, while the absorption intensity exhibited a decrease following 5-h incubation, the Sb-PDA compound’s stability remained suitable for cellular experimental applications.

### 3.4 Cytocompatibility, cellular interactions, and NIR effects of Sb-PDA

**FIGURE 4:**
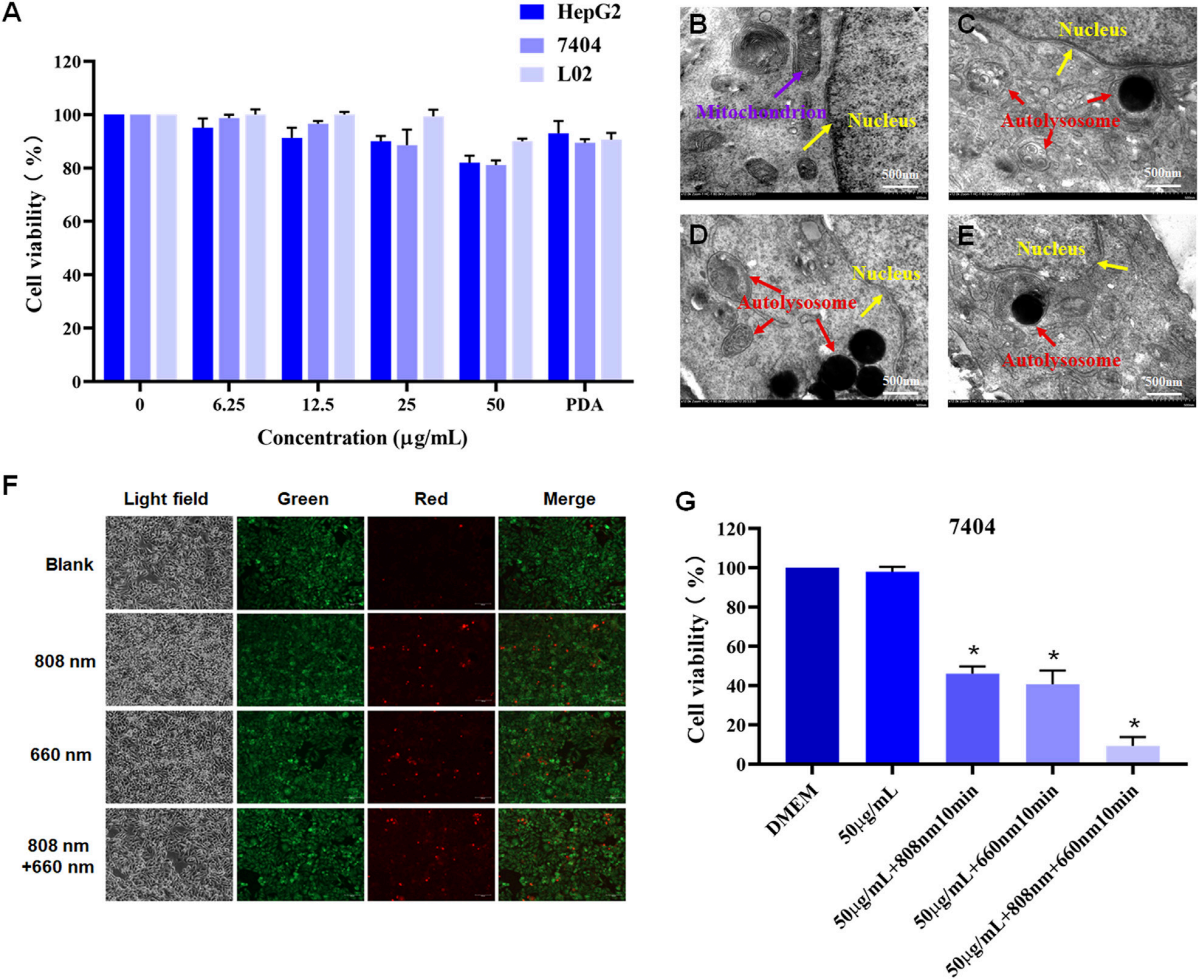
Cytocompatibility, cellular interactions, and NIR optical effects of Sb-PDA. **(A) **The cytotoxicity of Sb-PDA on HepG2, BEL-7404 and L-O2 cells. **(B–E)** Transmission electron microscopy of cellular substructures. Where **(B)** Blank (with material); **(C)** 808 nm laser treatment (1.5 W/cm^2^ 808 nm near-infrared irradiation for 10 min); **(D)** 660 nm laser treatment (0.5 W/cm^2^ 660 nm laser irradiation for 10 min); **(E)** 660 nm + 808 nm laser treatment (1.5 W/cm^2^ 808 nm and 0.5 W/cm^2^ 660 nm laser separately irradiated for 10 min each). **(F)** Cell staining of BEL-7404 cells under different laser treatments. **(G)** Cell viability of BEL-7404 cells after different laser treatments, *p < 0.05.

The CCK-8 assay demonstrated low cytotoxicity of Sb-PDA against normal hepatocytes (L-O2) and hepatoma cells (BEL-7404/HepG2) (survival rate >85%, Figure 4A), meeting the biocompatibility requirements for nano-phototherapeutic materials (Xiong et al., 2025). TEM revealed intact cellular structures in the untreated group (mitochondrial cristae clearly visible, Figure 4B), while the laser-treated groups exhibited autolysosome proliferation, Sb-PDA-containing secondary lysosomes, and vesicular structures encapsulating contents (Figures 4C–E). Fluorescence staining showed predominant green fluorescence in the untreated group (viable cells), red fluorescence (apoptotic/necrotic cells) in single-wavelength (660 nm or 808 nm) laser groups, and significantly increased red fluorescence in the dual-wavelength (660 nm + 808 nm) group (Figure 4F). Treatments with 50 μg/mL Sb-PDA combined with laser irradiation (660 nm/0.5W/cm^2^, 808 nm/1.5W/cm^2^, or dual-wavelength exposure for 10 min) all significantly reduced cell viability (*p* < 0.05, Figure 4G).

### 3.5 Necrosis, apoptosis and cycle analysis of BEL-7404 cells under different treatments

**FIGURE 5:**
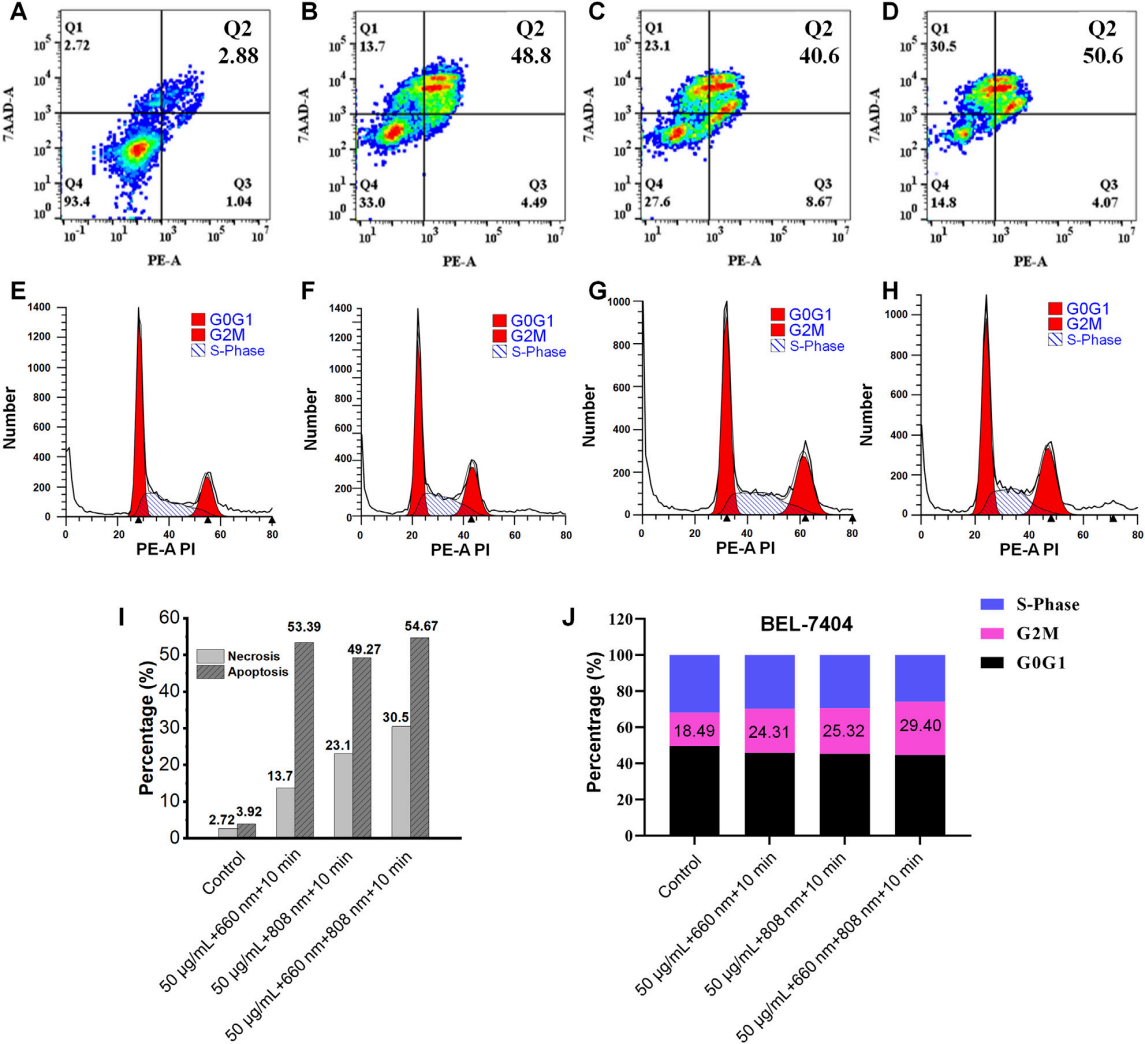
Necrosis, apoptosis and cycle analysis of BEL-7404 cells under different treatments. **(A–D)** Necrosis and apoptosis. **(E–H)** Cycle analysis. **(I)** Percentage of BEL-7404 cells in different phases of the cell necrosis and apoptosis. **(J)** Percentage of BEL-7404 cells in different phases of the cell cycle. Where **(A,E)** Blank (with material); **(B,F)** 808 nm laser treatment (1.5 W/cm^2^ 808 nm near-infrared irradiation for 10 min); **(C,G)** 660 nm laser treatment (0.5 W/cm^2^ 660 nm laser irradiation for 10 min); **(D,H)** 660 nm + 808 nm laser treatment (1.5 W/cm^2^ 808 nm and 0.5 W/cm^2^ 660 nm laser separately irradiated for 10 min each).

Flow cytometry results indicate the following apoptosis and cell cycle changes in BEL-7404 cells during phototherapy: compared to the non-irradiated group (necrosis rate 2.72%/apoptosis rate 3.92%), necrosis and apoptosis rates significantly increased after laser treatments (660 nm group: 13.7%/53.39%; 808 nm group: 23.1%/49.27%; combined group: 30.5%/54.67%, Figures 5A–D,I). Cell cycle analysis revealed a G2/M phase blockage tendency (control group 18.49% vs 660 nm group 24.31% vs 808 nm group 25.32% vs combined group 29.40%, Figures 5E–H,J). These results confirm that Sb-PDA composites effectively inhibit hepatoma cells through combined PTT/PDT via pathways of Apoptosis, Necrosis, Phase arrest, and phase arrest.

## 4 Conclusion

In summary, we employed a ligand-guided growth strategy to modulate the size of Sb NPs for tuning their absorption peak positions, successfully synthesizing Sb NPs with PTCE and biocompatibility. Experimental results confirmed that size modulation (40 nm–70 nm) induced a LSPR effect, causing UV-Vis absorption peak red-shift from 520 nm to 810 nm. Under 808 nm laser excitation, the PTCE reached 59.3%, while 660 nm laser excitation generated ROS, indicating a foundation for PDT. PDA modification yielded Sb-PDA composites that demonstrated low cytotoxicity in L-O2, BEL-7404, and HepG2 cells. *In vitro* anti-tumor experiments using BEL-7404 cells revealed that 660 nm/808 nm laser irradiation could inhibit proliferation, promote apoptosis, and induce G2/M phase blockage tendency, with combined irradiation exhibiting more significant effects. In conclusion, Sb-PDA shows preliminary potential for synergistic PTT/PDT, but its *in vivo* efficacy and mechanisms at the animal level require further exploration. LSPR-induced Sb-based nanomedicine may unlock diverse biomedical applications of semimetals.

## Data Availability

The original contributions presented in the study are included in the article/Supplementary Material, further inquiries can be directed to the corresponding authors.
