# Supplementary material for: Regulating the size of antimony nanoparticles to enhance the photo-response in the near-infrared region and anti-hepatoma cell activity
Source: Front Bioeng Biotechnol. 2025 Sep 5;13:1656253. doi: 10.3389/fbioe.2025.1656253 (PMC12446305; doi:10.3389/fbioe.2025.1656253)
Supplement: Supplementary file 1 [file DataSheet1.pdf]

![frontiers]

Supplementary Material

Regulating the Size of Antimony Nanoparticles to Enhance the Photo-response in the Near-infrared Region and Anti- hepatoma Cell Activity

Lingling Huang[1†], Yimin Gong[2,3,4†], Zhijian Chen[5,6†], Yanjun Tan[2,3], Qian Gao[2,3], Yilei Wang[2,3], Yuyu Gao[2,3], Wanting Cheng[2,3], Weiyuan Liang[2,3*], Xiaoli Yang[1, 2,3*]

*** Correspondence:**

Xiaoli Yang: cncsyxl@126.com
Weiyuan Liang: lwydoct2020@163.com

1 Supplementary Figures

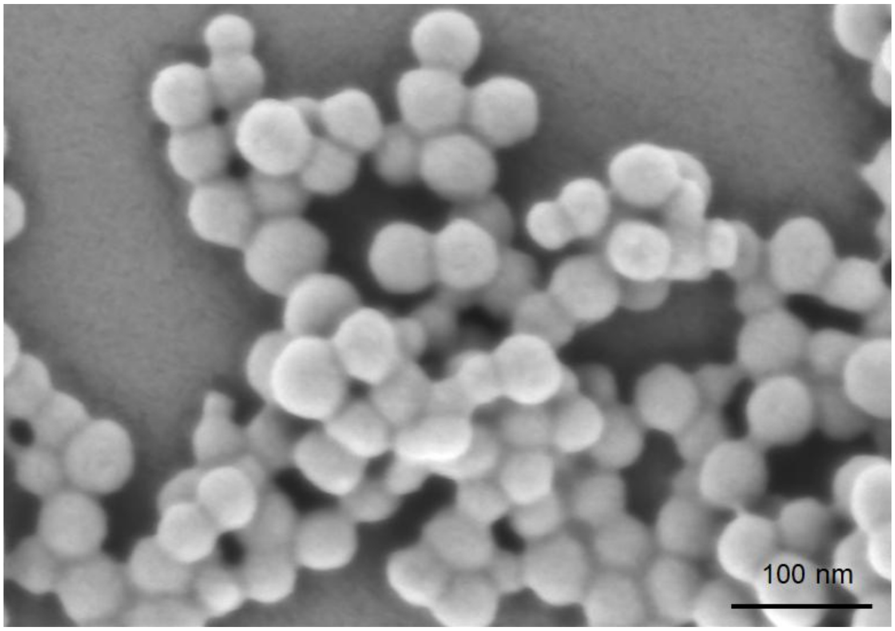

Supplementary Figure S1. SEM images of Sb NPs.

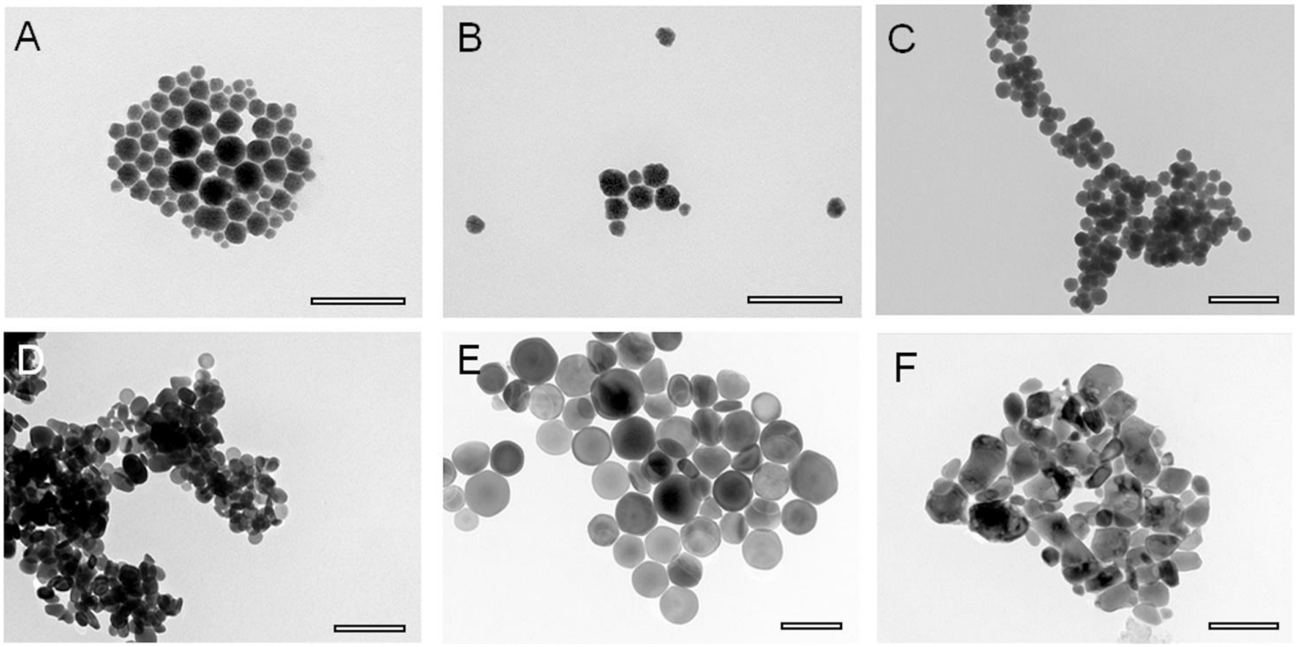

Supplementary Figure S2. Time-resolved TEM morphologies of Sb NPs. Morphologies of Sb NPs after reduced reaction for (A) 10 seconds; (B) 30 seconds; (C) 180 seconds; (D) 10 min; (E) 20 min; (F) 30 min, respectively. Scale bar: 200 nm.

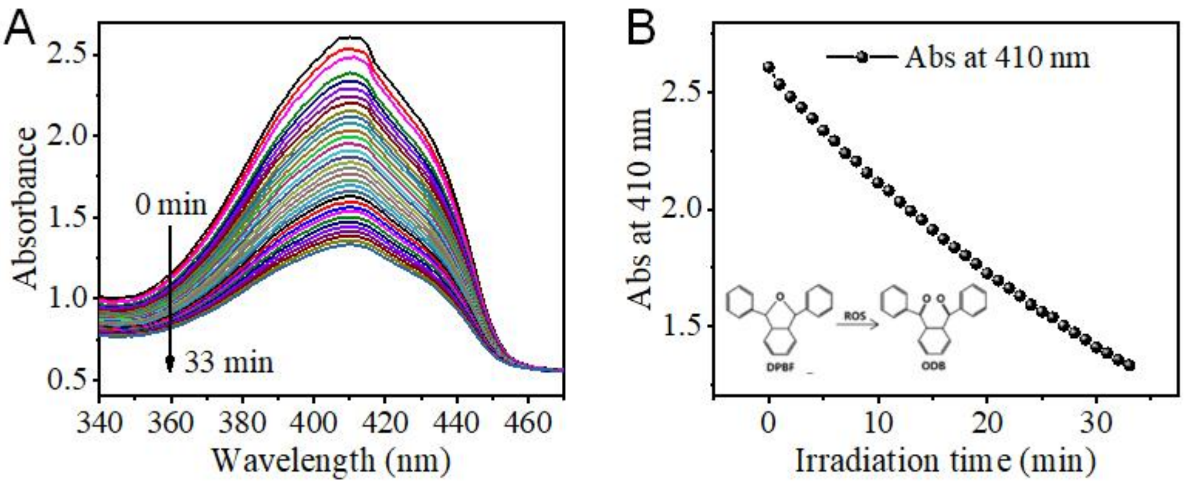

Supplementary Figure S3. UV absorbance spectra at 410 nm of DPBF in the solution of Sb NPs.

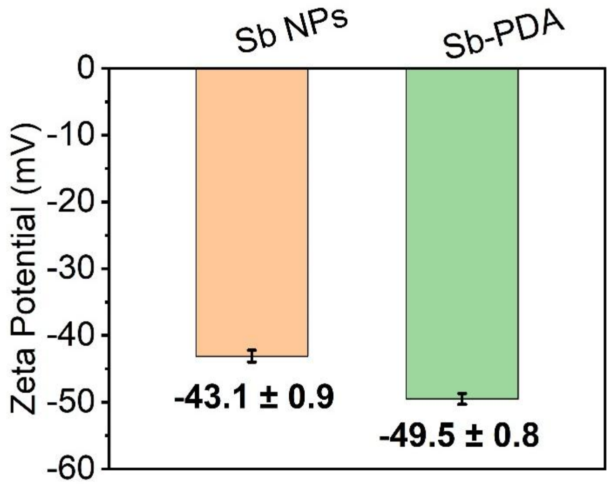

Supplementary Figure S4. Zeta-potential of Sb NPs and Sb-PDA.

Supplementary Table S1. Comparison of photothermal conversion efficiency (PTCE) from this research with those from previously published works on metallic and semiconductor nanomaterials for cancer treatment.

Materials	Laser irradiation	Photothermal conversion efficiency (PTCE)	Ref.
2D GeP nanosheets	808 nm	68.6%	[1]
Antimony nanopolyhedrons	808 nm	62.1%	[2]
Au nanoparticles	808 nm	61%	[3]
Antimony nanoparticles (Sb-PDA)	808 nm	59.3%	This work
Antimonene Quantum Dots	808 nm	45.5%	[4]
UCNPs@$AgBiS_2$ core-shell nanoparticles	808 nm	45%	[5]
Sb–THPP–PEG nanosheets	808 nm	44.6%	[6]
Black phosphorus nanosheets	808 nm	38.8%	[7]
Cr-based nanoparticles (Cr-PDA)	808 nm	37.4%	[8]
Nb_2C nanosheets (Mxene)	808 nm	36.4%	[9]
L-cysteine modified MoS_2	808 nm	35%	[10]

Reference

[1] X. Ren, W. Liu, H. Zhou, et al. Biodegradable 2D GeP nanosheets with high photothermal conversion efficiency for multimodal cancer theranostics[J]. Chemical Engineering Journal, 2022, 431: 134176.

[2] Y. Chen, M. Wang, K. Zheng, et al. Antimony Nanopolyhedrons with Tunable Localized Surface Plasmon Resonances for Highly Effective Photoacoustic-Imaging-Guided Synergistic Photothermal/Immunotherapy[J]. Advanced Materials, 2021, 33(18): 2100039.

[3] J. Depciuch, M. Stec, A. Maximienko, et al. Size-dependent theoretical and experimental photothermal conversion efficiency of spherical gold nanoparticles[J]. Photodiagnosis and Photodynamic Therapy, 2022, 39: 102979.

[4] W. Tao, X. Ji, X. Xu, et al. Antimonene Quantum Dots: Synthesis and Application as Near-Infrared Photothermal Agents for Effective Cancer Therapy[J]. Angewandte Chemie International Edition, 2017, 56(39): 11896-11900.

[5] Z. Chu, T. Tian, Z. Tao, et al. Upconversion nanoparticles@AgBiS2 core-shell nanoparticles with cancer-cell-specific cytotoxicity for combined photothermal and photodynamic therapy of cancers[J]. Bioactive Materials, 2022, 17: 71-80.

[6] Y. Kang, Z. Li, Y. Yang, et al. Antimonene Nanosheets-Based Z-Scheme Heterostructure with Enhanced Reactive Oxygen Species Generation and Photothermal Conversion Efficiency for Photonic Therapy of Cancer[J]. Advanced Healthcare Materials, 2021, 10(3): 2001835.

[7] M. Qiu, D. Wang, W. Liang, et al. Novel concept of the smart NIR-light–controlled drug release of black phosphorus nanostructure for cancer therapy[J]. Proceedings of the National Academy of Sciences, 2018, 115(3): 501.

[8] Q. Liu, Z. Sun, Y. Duo, et al. Chromium Nanoparticles Improve Macrophage and T Cell Infiltration for Cancer Immunotherapy[J]. ACS Materials Letters, 2023, 5(6): 1738-1751.

[9] H. Lin, S. Gao, C. Dai, et al. A Two-Dimensional Biodegradable Niobium Carbide (MXene) for Photothermal Tumor Eradication in NIR-I and NIR-II Biowindows[J]. Journal of the American Chemical Society, 2017, 139(45): 16235-16247.

[10] L. Ding, Y. Chang, P. Yang, et al. Facile synthesis of biocompatible L-cysteine-modified MoS2 nanospheres with high photothermal conversion efficiency for photothermal therapy of tumor[J]. Materials Science and Engineering: C, 2020, 117: 111371.
